# Bilateral Choroidal Detachment Following Pseudophakic Cystoid Macular Edema Treatment with Oral Acetazolamide

**DOI:** 10.3390/life15050811

**Published:** 2025-05-19

**Authors:** Agnieszka Kudasiewicz-Kardaszewska, Małgorzata Ozimek, Tomasz Urbański, Sławomir Cisiecki

**Affiliations:** 1 Prof. Zagórski Eye Surgery Center, OCHO Medical Group, Batorego 88, 33-300 Nowy Sącz, Poland; malgorzata.anna.ozimek@gmail.com (M.O.); turbanski@ocho.pl (T.U.); 2 Prof. Zagórski Eye Surgery Center, OCHO Medical Group, Kasztanowa 6, 24-150 Nałęczów, Poland; cisieckislawomir@gmail.com; 3 Department Ophthalmology, Jonscher Public Hospital, Milionowa 14, 93-113 Łódź, Poland

**Keywords:** choroidal effusion, choroidal detachment, carbonic anhydrase inhibitors (CAI), pseudophakic cystoid macular edema (PCME)

## Abstract

Aim: This case report presents an unusual instance of bilateral choroidal effusion following the oral administration of acetazolamide for the treatment of pseudophakic cystoid macular edema (PCME). Case Presentation: An 87-year-old Caucasian man experienced sudden, painless vision loss in both eyes several days after beginning treatment for PCME in his left eye. He had undergone uncomplicated cataract surgery in both eyes two months earlier. The treatment regimen included oral acetazolamide (250 mg twice daily) and topical pranoprofen, a nonsteroidal anti-inflammatory drug (NSAID). One week after the initiation of acetazolamide treatment, the patient suffered a marked decline in visual acuity. Bilateral choroidal effusion was diagnosed. Prompt discontinuation of acetazolamide and initiation of topical dexamethasone (1% hourly) and atropine (1% twice daily) resulted in rapid clinical improvement. Conclusions: Carbonic anhydrase inhibitors (CAIs) such as acetazolamide, although commonly used to manage intraocular pressure, can cause choroidal effusion—a rare but potentially sight-threatening complication. Ophthalmologists should exercise caution, particularly in elderly patients, and be alert to early signs of this adverse effect. Early diagnosis and prompt management are essential to prevent permanent visual damage. To our knowledge, this is the first reported case of bilateral choroidal detachment associated with acetazolamide in the context of PCME.

## 1. Introduction

Choroidal effusion syndrome is a rare but potentially vision-threatening condition characterized by the accumulation of fluid in the suprachoroidal space, leading to serous choroidal detachment [1,2]. This process may result in secondary complications such as severe hypotony and retinal detachment. The pathophysiology involves increased choroidal vascular permeability, impaired fluid outflow, or changes in hydrostatic and oncotic pressure. Known triggers include systemic conditions (e.g., hypertension), ocular diseases (e.g., central serous chorioretinopathy), ocular surgeries (e.g., glaucoma filtering procedures), and certain medications, particularly sulfonamide derivatives [3,4,5].

Acetazolamide, a widely used carbonic anhydrase inhibitor (CAI), effectively manages intraocular pressure and tissue edema. However, it has been associated with choroidal effusion in rare cases [6]. Although the precise mechanism remains unclear, it likely involves the disruption of choroidal vascular homeostasis.

This case report describes a unique instance of bilateral choroidal effusion following the administration of oral acetazolamide for the treatment of pseudophakic cystoid macular edema (PCME), also known as Irvine–Gass Syndrome.

## 2. Case Report

**Figure 1:**
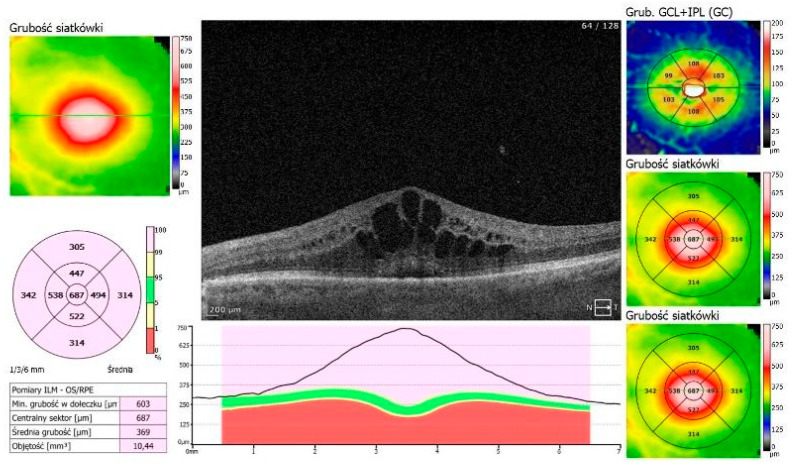
PCME—Irvin–Gass Syndrome in the LE. Best corrected visual acuity (BCVA) = 0.4.

An 87-year-old Caucasian male was admitted to our clinic with sudden, painless vision loss of his left eye. His medical history was unremarkable except for uneventful cataract surgery in both eyes two months prior. Two months postoperatively, he developed macular edema in the left eye, which was confirmed on optical coherence tomography (OCT, SOCT-Copernicus, Revo NX, Software version 11.5.1, Optopol Technology Ltd., Zawiercie, Poland) (Figure 1). Treatment for PCME was initiated with topical pranoprofen and oral acetazolamide (250 mg twice a day).

**Figure 2:**
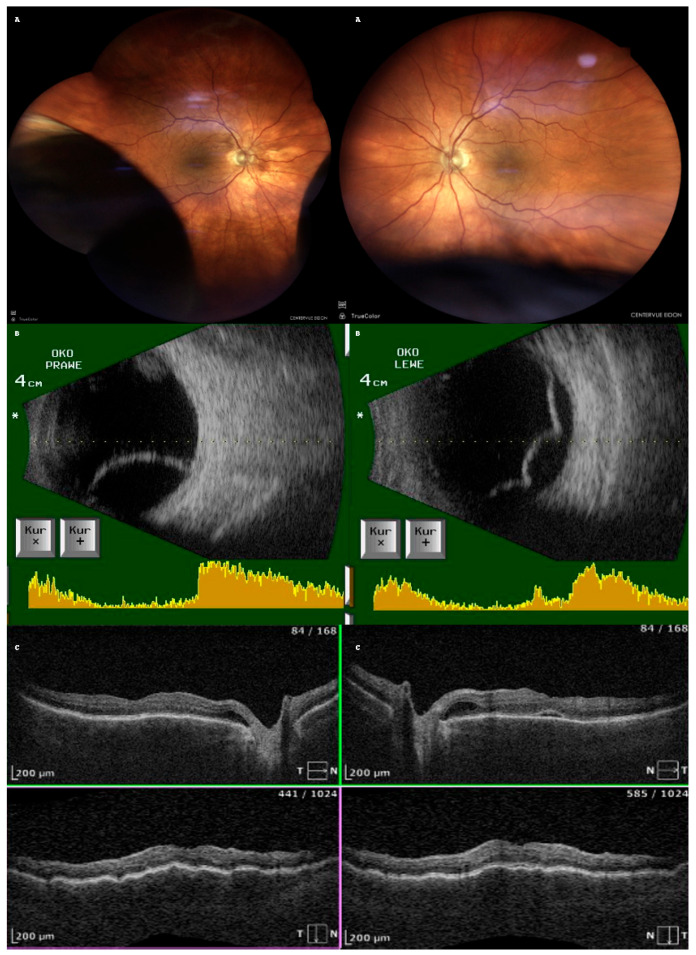
Imaging of choroidal effusion in both eyes: (**A**): Fundus photographs showing bullous choroidal detachment; (**B**): ultrasonography showing more pronounced detachment in the right eye; A scan is depicted on the bottom of the B scan (yellow diagram), top of the ultrasound probe is indicated as (*) (**C**): OCT showing choroidal folds and resolution of PCME in the left eye.

After one week of treatment for PCME, the patient returned with a bilateral vision decline. Fundoscopy revealed bilateral, infero-temporal and infero-nasal choroidal detachment (fundus photo with the use of confocal scanner Eidon, CenterVue, ICare Finland Oy, Vantaa, Finland); Figure 2A. Ultrasound examination confirmed bullous choroidal detachments (Ultrasound B-scan, Pirop, Echo-SonSA, Puławy, Poland) in both eyes (Figure 2B). OCT scans showed subretinal fluid (SRF) and choroidal folds in both eyes but complete resolution of the macular edema in the LE (Figure 2C). The intraocular pressure (IOP) measured with Tono-Pen AVIA (Reichert Technologies, Reichert Inc., Depew, NY, USA) was 16 mmHg (RE) and 14 mmHg (LE).

Ophthalmological examination details are depicted in Table 1.

**Figure 3:**
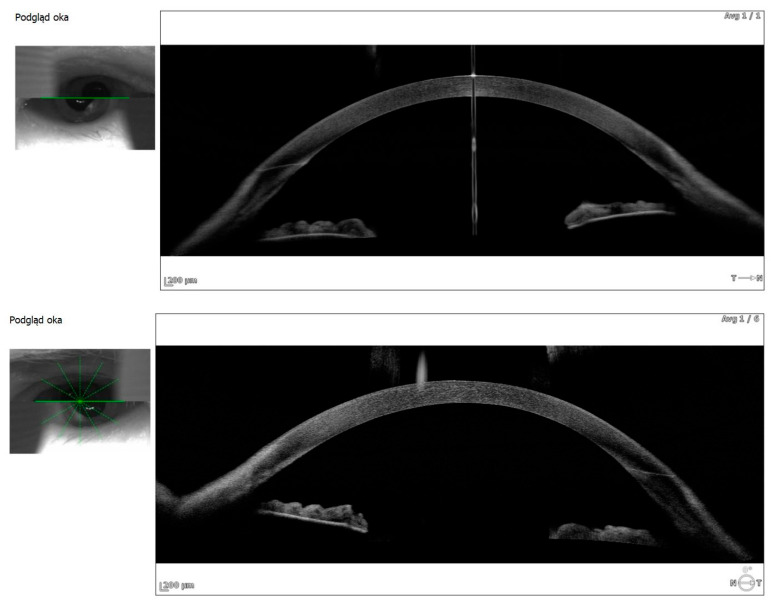
Anterior segment OCT—iris plateau configuration RLE (right eye: upper image, left eye: lower image).

Anterior segment OCT revealed iris plateau configuration, a possible contributing factor to the choroidal effusion (Figure 3).

Acetazolamide was discontinued immediately. Treatment included topical dexamethasone 0.1% every two hours, atropine 1% twice daily, and bromfenac twice daily. Significant improvement was observed within eigh days. Best corrected visual acuity (BCVA) improved to 0.5 Snellen (RE) and 0.6 Snellen (LE). OCT showed resolution of choroidal effusion and almost complete resorption of subretinal fluid (Figure 2).

At 29 days, the patient was stable, BCVA was 0.7 Snellen (RE) and 0.6 Snellen (LE), and treatment was discontinued.

**Figure 4:**
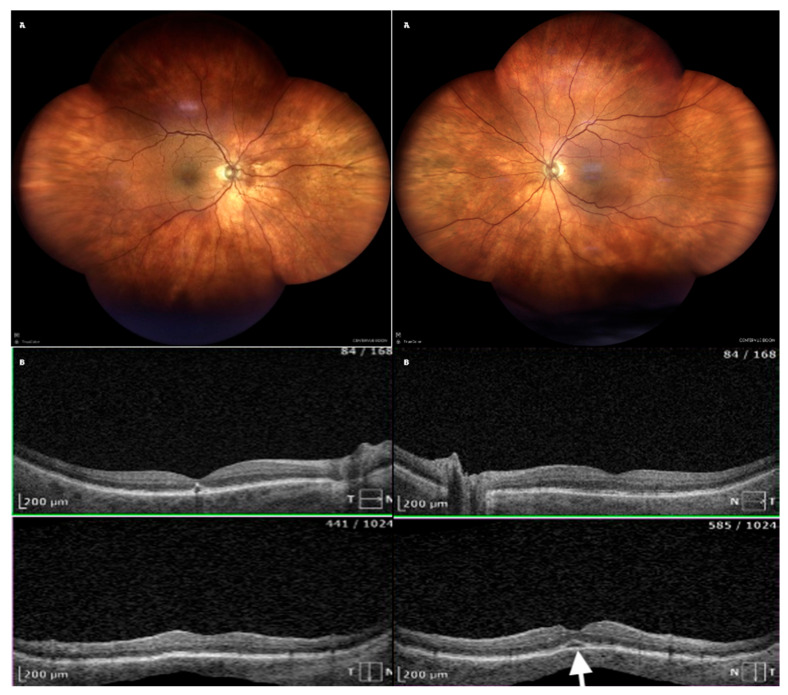
Resolution of choroidal effusion at day 8. (**A**) Fundus photograph—choroid and retina attached, bullous detachment disappeared; (**B**) OCT—no folds, remnants of the subretinal fluid in the LE (arrow).

Following the immediate discontinuation of acetazolamide and the initiation of topical therapy, the patient showed rapid improvement. Within eight days, visual acuity significantly improved. Imaging confirmed the complete resolution of choroidal effusion (Table 2 and Figure 4). At a follow-up visit on Day 29, the patient remained stable, with no recurrence of symptoms (Table 3).

## 3. Discussion 

Choroidal effusion is a complex and multifactorial condition marked by the accumulation of fluid in the suprachoroidal space, often resulting in serous choroidal detachment. If not diagnosed and treated promptly, it may cause significant and potentially irreversible visual impairment. Although rare, choroidal effusion has been associated with systemic conditions (e.g., hypertension and altitude sickness), ocular surgeries (e.g., glaucoma procedures), and pharmacological agents, including carbonic anhydrase inhibitors (CAIs) [2].

Acetazolamide, a widely used carbonic anhydrase inhibitor (CAI), is commonly prescribed for glaucoma, idiopathic intracranial hypertension, and macular edema. It is commonly used for altitude sickness [7]. Acetazolamide is routinely prescribed in addition to non- steroidal anti-inflammatory drugs (NSAIDs) in PCME. Despite its effectiveness in reducing intraocular pressure and retinal fluid, acetazolamide has been linked to rare adverse effects, including choroidal effusion and secondary angle-closure glaucoma [6,8]. This report describes a rare instance of bilateral choroidal effusion in an elderly patient treated with oral acetazolamide for PCME.

### 3.1. Proposed Pathophysiological Mechanisms of Acetazolamide-Induced Choroidal Effusion 

Several mechanisms have been proposed to explain the pathogenesis of acetazolamide-induced choroidal effusion. These mechanisms likely act in combination, leading to the accumulation of fluid in the suprachoroidal space.

### 3.2. Increased Choroidal Vascular Permeability and Endothelial Dysfunction 

Acetazolamide, a sulfonamide derivative, may compromise the endothelial integrity of choroidal blood vessels, leading to plasma leakage and fluid accumulation in the suprachoroidal space [9].

### 3.3. Alteration in Hydrostatic and Osmotic Pressure 

As a diuretic, acetazolamide reduces systemic fluid retention by inhibiting carbonic anhydrase activity. This may decrease systemic venous pressure, disturbing the hydrostatic balance between intraocular and extraocular compartments. The resulting pressure gradient may promote fluid accumulation in the choroidal space. Additionally, acetazolamide’s suppression of aqueous humor production may lead to a rapid drop in intraocular pressure (IOP), potentially causing ciliochoroidal detachment [4].

### 3.4. Direct Effect on the Ciliary Body and Choroidal Blood Flow 

The inhibition of carbonic anhydrase isoenzymes within the ciliary body leads to decreased aqueous humor secretion, but it may also impact the fluid dynamics within the choroidal circulation. Carbonic anhydrase plays an important role in the regulation of pH and ionic balance in ocular tissues. Its inhibition may disrupt the normal function of the choroidal capillaries, leading to increased extravasation of fluid into the suprachoroidal space [1].

### 3.5. Idiosyncratic Drug Reaction and Immune-Mediated Response 

Some patients may experience an idiosyncratic hypersensitivity reaction to carbonic anhydrase inhibitors. This reaction is defined as an abnormal susceptibility to a drug peculiar to the individual [8,10]. This phenomenon has been observed with both systemic (e.g., acetazolamide) and topical (e.g., dorzolamide and brinzolamide) CAIs [11]. These reactions may cause uveal effusion, anterior segment inflammation, and choroidal detachment, particularly in elderly individuals with altered pharmacokinetics and decreased drug clearance [12].

## 4. Review of Previous Literature and Comparisons 

Choroidal effusion associated with carbonic anhydrase inhibitors (CAIs) has been documented in previous case reports and studies, supporting the hypothesis that CAIs can trigger fluid accumulation in the suprachoroidal space. There are examples presented below. There was a case report in which a 76-year-old patient developed bilateral angle-closure glaucoma and extensive choroidal detachment following oral acetazolamide administration after routine cataract surgery. The condition improved rapidly upon discontinuation of acetazolamide and initiation of high-dose intravenous steroid therapy. This case highlights the importance of early steroid intervention in CAI-induced choroidal effusion [13].

An echographic study was conducted evaluating the incidence of uveal effusion after cataract surgery. The findings indicated that the postoperative combination of oral acetazolamide and topical pilocarpine gel significantly increased the risk of choroidal effusion, suggesting that certain pharmacological combinations may exacerbate this condition [14]. A case of a 60-year-old male with plateau iris configuration who developed bilateral ciliochoroidal effusion syndrome after acetazolamide use was described. The patient presented with a myopic shift, elevated intraocular pressure, and shallow anterior chambers. Upon discontinuation of acetazolamide and the initiation of topical therapy, the choroidal effusion resolved. This case demonstrates how predisposing anatomical factors may contribute to the severity of acetazolamide-induced choroidal detachment [15]. Liyanage et al. reported two cases of uveal effusion following acetazolamide administration, one after cataract surgery and another following prophylactic treatment for altitude sickness. In both cases, timely discontinuation of the drug led to complete resolution of symptoms, underscoring the reversibility of CAI-induced effusions with appropriate management [16].

## 5. Clinical Implications and Recommendations for Management 

Awareness of acetazolamide-induced choroidal effusion is vital for ophthalmologists, especially when prescribing this medication in postoperative settings or in elderly and anatomically predisposed patients [8,15,17]. Elderly individuals and those with a history of choroidal detachment, angle-closure glaucoma, or uveal effusion should be carefully monitored when prescribed acetazolamide or other CAIs. Regular follow-up with ultrasonography and optical coherence tomography (OCT) can aid in the early detection of subclinical choroidal effusion before symptomatic vision loss occurs [12,14].

The combination of acetazolamide with miotics like pilocarpine may increase the risk of uveal effusion and secondary angle closure [14]. Alternative treatments for postoperative macular edema should be considered for patients with known risk factors [1].

If choroidal effusion is suspected, acetazolamide should be discontinued immediately to prevent worsening of the condition. Alternative anti-inflammatory therapy, including topical corticosteroids (dexamethasone) and cycloplegics (atropine), should be initiated to reduce inflammation and promote fluid resolution [2].

In cases with significant visual impairment or extensive choroidal detachment, systemic or periocular corticosteroids may be beneficial in hastening resolution [1].

## 6. Conclusions 

This case demonstrates that CAIs can rarely lead to choroidal effusion—a potentially sight-threatening complication, particularly in elderly or anatomically predisposed patients.

To the best of our knowledge, this is the first reported case of bilateral choroidal effusion following oral acetazolamide treatment specifically for PCME. Prompt discontinuation of the CAI and initiation of topical corticosteroids and mydriatics resulted in complete clinical and anatomical resolution.

Further research is warranted to identify risk factors and underlying mechanisms, including idiosyncratic or hypersensitivity reactions, that may predispose certain patients to CAI-induced choroidal effusion. Improved understanding may help prevent such adverse events in the postoperative course of susceptible individuals.

Elderly patients receiving acetazolamide after cataract surgery are potentially at risk for bilateral choroidal effusion. Careful monitoring, immediate cessation of the drug, and prompt anti-inflammatory therapy can ensure complete recovery.

In case of PCME in elderly patients, topical therapy with steroids and NSAIDs could be considered first prior to the initiation of systemic oral acetazolamide therapy.

## 7. Plain Language Summary

This case report describes an 87-year-old man who suddenly lost his vision in both eyes after taking a medication called acetazolamide for a common eye condition known as pseudophakic cystoid macular edema (PCME), which can occur after cataract surgery. The drug was meant to reduce swelling in the eye, but it caused an unexpected problem—fluid built up behind both of his eyes, a condition called choroidal effusion.

The patient was treated with acetazolamide and anti-inflammatory eye drops due to PCME (Irvin–Gass syndrome) in the left eye. About a week after starting the medication, he began to lose his vision. Eye scans showed that fluid had collected under the choroid in both eyes. The doctors immediately stopped the acetazolamide and started treatment with steroids and atropine eye drops. Within eight days, his vision improved significantly, and the fluid had cleared up.

This report highlights a rare but serious side effect of acetazolamide. While it is commonly used to lower eye pressure or treat swelling, it can sometimes cause fluid under the choroid, leading to its detachment. Older adults may be more at risk for this side effect. Recognizing the problem early and stopping the medication is important to prevent permanent vision loss.

This appears to be the first reported case of both eyes being affected by choroidal effusion after using acetazolamide to treat PCME. It also indicates that in case of PCME, particularly in elderly patients, topical therapy with steroids and NSAIDs could be considered first prior to initiation of systemic oral acetazolamide therapy.

## Figures and Tables

**Table 1:** Summary of findings on Day 1 (onset of choroidal effusion).

Day 1	
Chief Complaint (CC)	acute, painless bilateral vision deterioration
BCVA	BCVA RE = 0.3 cc − 1.5 sph, −1.25 cyl ax 88 BCVA LE = 0.4 cc + 0.25 sph, −1.0 cyl ax 107
Anterior Segment	Moderately shallow anterior chambers in both eyes (confirmed on anterior segment OCT)
Posterior Segment Figure 2A	Choroidal effusion in the inferior–temporal and inferior–nasal quadrants in both eyes
USG ScanB Figure 2B	Inferior–temporal and inferior–nasal choroidal detachment, more pronounced in RE
OCT Figure 2C	No cystoid macular edema in the left eye; residual subretinal fluid (SRF) and choroidal folds in both eyes; fluid next to the optic disk and macular choroidal folds in both eyes
IOP	RE 16 mmHg LE 14 mmHg
Treatment	Immediate discontinuation of acetazolamide; 0.1% dexamethasone eye drops every two hours—both eyes; 1% Atropine eye drops twice a day—both eyes; Bromfenac twice a day—left eye.

**Table 2:** Case presentation summary at day 8—following one week of treatment for choroidal effusion.

Day 8	
CC	Visual acuity improvement
BCVA	BCVA RE = 0.5 cc 0 sph, −1.5 cyl ax 88 BCVA LE = 0.6 + 0.5 sph, −1.0 cyl ax 107
Slit lamp exam and fundoscopy	AC deepened; Complete resolution of choroidal effusion
OCT	Residual submacular subretinal fluid persisted in the left eye, fluid next to the optic disk resolved, and choroidal folds disappeared
IOP	RE 16 mmHg, LE 16 mmHg
Treatment	0.1% dexamethasone eye drops 4 times a day to both eyes for 7 days; topical bromfenac 2xdaily—both eyes; 1% atropine eye drops were discontinued.

**Table 3:** Follow-up visit at Day 29.

Day 29	
CC	No complains
BCVA	BCVA RE = 0.7 cc 0 sph, −1.5 cyl ax 90, cyl ax 88 BCVA LE = 0.6 + 0.5 sph, −1.0 cyl ax 107
Treatment	All medications discontinued

## Data Availability

The authors declare that all data supporting the report are available upon request to the corresponding author.
